# Description of a Plant Yielding Asa Fœtida

**Published:** 1786

**Authors:** John Hope


XVI. *Description of a Plant yielding Asa Fœtida. In a Letter from* John Hope, *M. D. F. R. S. to Sir* Joseph Banks, *Bart. P. R. S.* Vide *Philosophical Transactions of the Royal Society of London,* Vol. lxxv, *for the year* 1785: 4to London, 1785.

ALTHOUGH Asa Fœtida has, for many centuries, formed a part of the Materia Medica, there was no satisfactory account of the plant from which it was obtained, till Kæmpfer, in the year 1712, described and delineated it in his *Amoenitates Exoticæ*. The plant which is the subject of the present paper, was raised, it seems, in the botanic garden at Edinburgh, from a root sent to Dr. Hope from St. Petersburgh, where it had been cultivated by Professor Pallas, from seed procured from Persia. Every part of the plant when wounded, poured out a rich milky juice, resembling in smell and taste asa fœtida; and, at times, a smell resembling garlick, such as a faint impregnation of asa fœtida yields, was perceivable, we are told, at the distance of several feet.

As the plant grows in the open air, without protection, and even in an unfavourable season proproduced a good deal of feed; and as the juice feems to be of the fame nature with the officinal afa fœtida, our author thinks there is fome reafon to hope, that it may become an article of cultivation in this countiy, of no inconfiderable importance.

The following is Dr. Hope's botanical defcription of the plant:

"ASA FŒTIDA.

"PLANTA umbellifera, tripedalis, erecta,

" ramofa, glauca, flore luteo.

" *Radix* perennis.

" *Folia* radicalia fex, procumbentia, trilobo-

" ovata, multoties pinnatim divifa;

" foliolis incifis, fubacutis, fubdecur-

" rentibus; petiolo communi fuperne

" plano, linea elevata longitudinaliter

" per medium decurrente.

" *Caulis* bipedalis, erectus, teretiufculus, an-

" nuus, leviter ftriatus, glaber, nudus

" præter unam circa medium foliorum

" imperfectorum conjugationem; pe-

" tiolo membranaceo, concavo.

" *Rami* nudi, patuli; quorum tres inferi, al-

" terni, fuftinentur finguli folii imper-

" fecti petiolo membranaceo concavo.

 Quatuor

" Quatuor intermedii verticillati sunt.

" Supremi ex apice caulis octo, quo-

" rum interni erecti.

" Omnes hi rami summitate sustinent

" umbellam compositam sessilem ter-

" minalem, et præterea 3 — 6 ramulos

" externe positos, umbellas compositas

" ferentes.

" Hoc modo, rami inferiores sustinent 5,

" raro 6 ramulos; intermedii 3 vel 4;

" superiores 1 et 2.

" CAL. *Umbella universalis* radiis 20—30 constat.

" ——— *partialis* flosculis subsessilibus 10

" —20.

" *Umbella composita* sessilis convexo-plana.

" ——— ——— pedunculata hæmisphaerica.

" *Involucrum universale* nullum.

" ——— *partiale* nullum.

" *Perianthium proprium* vix notabile.

" COR. *Universalis* uniformis.

" ——— Flosculi umbellæ sessilis fertiles.

" ——— ——— pendunculatæ plerumque

" abortiunt.

" *propria* petalis quinque æqualibus, pla-

" nis, ovatis: primo patulis, dein re-

" flexis, apice ascendente.

" STAM.

"Stam. *Filamenta* 5, ſubulata, corolla lon-

"giora, incurvata. *Antheræ* ſubrotundæ.

"Pist. *Germen* turbinatum, inferum.

"*Styli* duo, reflexi.

"*Stigmata* apice incraſſata.

"Per. nullum: fructus oblongus, plano-

"compreſſus, utrinque 3 lineis elevatis

"notatus eſt.

"Sem. duo, oblonga, magna, utrinque plana,

"3 lineis elevatis notata.

"Planta odorem alliaceum diffundit. Fo-

"lia, rami, pedunculi, radix, truncus,

"ſecti ſuccum fundunt lacteum, ſa-

"pore et odore Aſæ fœtidæ."

This deſcription is accompanied with two engravings, which differ in many reſpects from the figure of the plant given by Kæmpfer. This circumſtance has induced the learned Preſident, to whom the paper is addreſſed, to add the following note to Dr. Hope's letter.

"Probably Kæmpfer's Aſa fœtida plant is a "different ſpecies from that deſcribed by Dr "Hope in this paper. Kæmpfer was himſelf "upon the mountains where the drug is col- "lected, and his fidelity in deſcribing as well "as delineating, has not hitherto been impeach- "ed. Sanguis Draconis, and ſome other gums, "are indifferently the produce of various ſpe-

"cies

"cies of plants; and why may not Afa fœtida "be fimilarly circumftanced? Jos. Banks."

